# Objective optical quality in eyes with customized selection of aspheric intraocular lens implantation

**DOI:** 10.1186/s12886-019-1162-6

**Published:** 2019-07-18

**Authors:** Qing-Qing Tan, Jia Lin, Jing Tian, Xuan Liao, Chang-Jun Lan

**Affiliations:** 1 Department of Ophthalmology, Affiliated Hospital of North Sichuan Medical College, 1 Mao Yuan Nan Road, Nanchong, 637000 Sichuan China; 2 Department of Ophthalmology and Optometry, North Sichuan Medical College, 1 Mao Yuan Nan Road, Nanchong, 637000 Sichuan China

**Keywords:** Cataract, Aspheric IOL, Customized, Higher-order aberrations, OQAS

## Abstract

**Background:**

To compare the postoperative optical quality in eyes with customized selection and random selection of aspheric intraocular lens (IOL) implantation.

**Methods:**

A prospective, nonrandomized study was implemented in adult cataract patients who underwent unilateral phacoemulsification with aspheric IOL implantation. Patients were allocated into two treatment groups: a customized group and a control group. In the customized group, the aspheric IOL selection was based on the corneal spherical aberration to enable the postoperative target ocular spherical aberration closest to zero; in the control group, the aspheric IOLs were chosen using a random strategy. Primary outcome measurements included the following objective optical quality assessments: higher-order aberrations obtained by a Hartmann-shack aberrometer at 4 mm and 6 mm pupil diameters; objective scatter index (OSI), modulation transfer function (MTF) cut-off, Strehl ratio (SR) and a simulated contrast visual acuity—optical quality analysis system value (OV) obtained by a double-pass system with a 4-mm aperture. Subjective visual acuity was measured as secondary outcome. All the patients were followed up for 3 months.

**Results:**

Eighty-four patients in the customized group and 78 patients in the control group were evaluated. There was no significant difference in postoperative visual acuity between the two groups (*P* > 0.05). Significantly less ocular higher-order aberrations were shown in the customized group (*P* < 0.05). No significant difference was shown in OSI, MTF cut-off, SR and OV between the two groups (*P* > 0.05).

**Conclusions:**

Although customized selection of aspheric IOL implantation showed less postoperative ocular aberrations, it performed similarly to random selection of aspheric IOL implantation in terms of postoperative visual acuity, simulated contrast visual acuity, intraocular scatter, modulation transfer function and Strehl ratio.

**Trial registration:**

Retrospectively registered on 07/06/2019. Registration number: ChiCTR1900024356.

## Background

To obtain optimal optical quality and visual performance for the pseudophakic eyes, reducing the higher-order aberrations (HOAs) is demanded. As one of the most important HOAs, spherical aberration (SA) has been paid much attention by researchers [1]. A human cornea has a SA of + 0.27 μm as the mean value at 6 mm pupil diameter (PD) [2], a spherical intraocular lens (IOL) has positive SA as well, thus it could worsen the postoperative ocular SA and then impair the retinal image quality; an aspheric IOL has a prolate aspheric surface that creates a zero or negative value of SA, which could totally or partially compensate the corneal positive SA, then reduce the postoperative ocular SA and therefore improve the visual performance [3]. Currently, there are several clinically available aspheric IOLs with different labeled SA values, such as the SA value of − 0.27 μm (AMO Tecnis ZCB00), − 0.20 μm (Alcon SN60WF IQ) and 0 μm (Bausch & Lomb Akreos Adapt AO). These different labeled SA designs aim to achieve optimal optical quality and visual performance by appropriately compensating preoperative corneal SA.

Wavefront aberrometers have been widely used in previous studies for assessing the optical quality by measuring the aberrations. However, they have a lack of information on quite higher-order aberrations and intraocular scatter [4]. In addition to aberrations, intraocular scatter is another important optical factor affecting visual performance. Optical quality analysis system (OQAS), based on the double-pass technique, provides information for both aberrations and intraocular scatter and produces a more accurate and complete description of the optical quality [4, 5]. OQAS provides parameters including objective scatter index (OSI), modulation transfer function (MTF) cut-off, Strehl ratio (SR), and a simulated contrast visual acuity—optical quality analysis system values (OV) at contrasts of 100, 20 and 9%, which allow comprehensive assessment of the optical quality of the human eyes [6]. It has been successfully used to evaluate ocular optical quality in pseudophakic eyes [7, 8] and exhibited good repeatability [9, 10].

Previous non-controlled studies reported that applying a customized selection of aspheric IOL implantation based on corneal SA could precisely reduce the postoperative ocular SA closest to zero [11, 12]. However, the current limited controlled studies merely measured SA or contrast sensitivity for assessing the visual quality, and there is still a lack of consensus whether optical quality following a customized selection with a target ocular SA of zero differs from that following a non-customized selection [13, 14]. In this study, we applied a random selection strategy in the control group, by which we aimed to replicate the aspheric IOL selection strategy that we have been using in actual clinical practice in the past. We hypothesized that customized selection creates an equal postoperative optical quality with a random selection of aspheric IOL implantation. Using a combination of Hartmann-shack aberrometer and double-pass instrument with a comprehensive and objective assessment, this study aimed to determine whether customized selection differs from random selection of aspheric IOL in postoperative optical quality.

## Methods

### Study design and participants

This prospective study was approved by the Institutional Review Board of Affiliated Hospital of North Sichuan Medical College. Adult cataract patients undergone unilateral phacoemulsification and IOL implantation from October 2015 to December 2016 in Affiliated Hospital of North Sichuan Medical College were enrolled. Informed consents were obtained from all patients. Exclusion criteria included corneal astigmatism> 1.00 diopters (D), irregular astigmatism and other ocular pathology that could potentially affect the postoperative vision, such as corneal pathology, uncontrolled glaucoma, existing retinal or optic neuropathy.

Totally 182 eyes of 182 patients were allocated into 2 treatment groups: the customized group and the control group. In the customized group, 92 eyes of 92 patients were implanted with an aspheric IOL selected based on corneal SA at 6 mm PD. The IOL allocation method in the customized group was adapted from Solomon’s study in 2010 [11]. For corneal SA < + 0.1 μm, Bausch & Lomb Akreos Adapt AO (with a labelled SA of 0 μm) was selected; for corneal SA ≥ + 0.1 μm but ≤ + 0.235 μm, Alcon SN60WF IQ was selected (with a labelled SA of − 0.20 μm); for corneal SA > + 0.235 μm, AMO Tecnis ZCB00 was selected (with a labelled SA of − 0.27 μm). In the control group, 90 eyes of 90 patients were implanted with 1 of 3 aspheric IOL types stated above using a random selection strategy.

### Surgical procedures

All operations were performed by the same experienced surgeon (CJL) and using the same phacoemulsification machine (Stellaris, Bausch & Lomb Co. Ltd., USA). A 2.8 mm clear corneal incision at the meridian of 135° was made, a continuous circular capsulorhexis was performed, followed by hydrodissection and phacoemulsification cataract extraction. Then, an aspheric IOL was implanted in the capsular bag.

### Outcome evaluation

All patients had a comprehensive ophthalmic examination before the surgery. The irregularities of the cornea and the IOL allocation in the customized group were determined by measuring the corneal topography and wavefront aberrations using a Hartmann-shack based wavefront analyzer (KR-1 W, Topcon Corp., Tokyo, Japan).

All postoperative evaluations were performed at 3 months after surgery, including routine examinations: refraction, uncorrected visual acuity (UCVA), best corrected visual acuity (BCVA), slit-lamp examination, funduscopy, and tonometry. Visual acuity was measured under photopic condition (85 cd/m^2^). Tilt and decentration of the IOLs in relation to the visual axis were assessed using a Scheimpflug imaging system (Pentacam, Oculus Inc., Wetzlar, Germany).

Meanwhile, wavefront aberrations were measured using the identical device as baseline measurements under mesopic condition (3 cd/m^2^). Aberrations were measured after dilation by instillation of a mixture of 0.5% tropicamide and 0.5% phenylephrine (Mydrin P, Santen Pharmaceutical Co., Osaka, Japan) in all eyes. Based on Zernike coefficients, SA (Z_4_^0^), root mean square (RMS) values of coma (Z_3_^− 1^, Z_3_^1^), trefoil (Z_3_^− 3^, Z_3_^3^) and total higher-order aberration (t-HOA, 3rd to 6th order aberrations) were analyzed for 4 mm PD and 6 mm PD respectively.

OQAS (OQAS II; Visiometrics SL, Terrassa, Spain) measurements were performed postoperatively in the same mesopic condition. All measurements were performed with a 4-mm aperture, which is a standard size used in clinical double-pass studies [15]. The following parameters were measured to quantify the optical quality: 1) OSI, defined as the ratio of the light of peripherally annular area versus that of the central peak in the acquired double-pass image, quantifies intraocular scatter [16]. The lower OSI values indicate better optical quality; 2) MTF cut-off, the cut-off point of the MTF curve on the x-axis that can be directly computed from the point spread function (PSF), denotes the highest spatial frequency at which the MTF reaches the lowest contrast of 1% [16]. The higher MTF cut-off values indicate better optical quality; 3) SR, the ratio of peak focal intensities in aberrated PSF and ideal PSF, indicates a perfect optical system when it is equal to 1 and an aberrated optical system when it is less than 1 [16]; 4) OVs (OV 100%, OV 20% and OV 9%), corresponding to three different spatial frequencies of the MTF values, describe optical quality for three contrast conditions that are commonly used in ophthalmology practice [16]. Specifically, OV 100% is directly related to the MTF cut-off frequency [17]. The OVs can be used to obtain more specific information about the behavior of the system at different contrasts, which may remain hidden when more global parameters that integrate the information along all available spatial frequencies are considered, such as the Strehl ratio [18].

### Statistical analysis

All data were analyzed using SPSS 20.0 software. A Shapiro-Wilk test was used for testing the normal distribution. Normal distributions were shown in all continuous data in both groups (*P* > 0.05). Continuous variables were expressed as Mean ± standard deviation (SD), dichotomous variables were expressed as percent (n%). An independent samples *t*-test was performed for comparisons between the two groups, and a contingency Chi-square test was performed for dichotomous data. A *P* value < 0.05 was considered statistically significant.

## Results

Eighty-four patients (38 women, 46 men) in the customized group and 78 patients (37 women, 41 men) in the control group were evaluated. The mean age was 65.26 ± 8.91 years old in the customized group and 67.84 ± 9.64 years old in the control group. Preoperative demographics, corneal astigmatism, corneal HOAs and IOL power showed no significant difference between the two groups (Table 1). In the customized group, Akreos Adapt AO IOLs were selected for 1 patient, whose preoperative corneal SA was 0.085 μm; SN60WF IQ IOLs were selected for 26 patients, whose mean preoperative corneal SA was 0.21 ± 0.05 μm; Tecnis ZCB00 IOLs were selected for 57 patients, whose mean preoperative corneal SA was 0.33 ± 0.08 μm. In the control group, Akreos Adapt AO, SN60WF IQ and Tecnis ZCB00 IOLs were selected for 27, 26 and 25 patients respectively. There was no intraoperative complication in any case. During follow-up, no tilt > 4° or decentration > 0.25 mm of any IOL was encountered, which were significantly lower than the clinically significant tilt and decentration cut-offs (7° and 0.4 mm, respectively) defined by Holladay et al. [19]. The differences in tilt and decentration between the two groups were not statistically significant (*P* > 0.05; independent *t*-test). The following patients were excluded: in the customized group, 1 patient had severe posterior capsular opacity, 1 patient had macular edema and 6 patients were lost to follow-up; in the control group, 2 patient had severe posterior capsular opacity, 10 patients were lost to follow-up.

**Table 1:** Demographics and baseline characteristics of the patients

	Customized group *N* = 84	Control group *N* = 78	*P* value
Sex (female, %)	45.2%	47.4%	0.78^a^
Laterality (surgery in the right eye, %)	46.4%	55.1%	0.27^a^
Age (Mean ± SD, year)	65.26 ± 8.91	67.84 ± 9.64	0.07^b^
Corneal astigmatism (Mean ± SD, D)	0.59 ± 0.21	0.52 ± 0.25	0.07^b^
IOL power (Mean ± SD, D)	19.91 ± 3.43	19.42 ± 3.36	0.36^b^
t-HOA (6 mm pupil)	0.69 ± 0.19	0.75 ± 0.25	0.15 ^b^
SA (6 mm pupil)	0.28 ± 0.07	0.29 ± 0.07	0.66 ^b^
Coma (6 mm pupil)	0.21 ± 0.10	0.23 ± 0.13	0.38 ^b^
Trefoil (6 mm pupil)	0.49 ± 0.18	0.52 ± 0.22	0.36 ^b^

*t-HOA* total higher-order aberration, *SA* spherical aberration, *SD* standard deviation, *D* diopter; ^a^: Chi-square test; ^b^: Independent t-test

### Visual acuity

There was no significant difference in postoperative UCVA (0.14 ± 0.16 LogMAR for the customized group vs 0.16 ± 0.14 LogMAR for the control group; *P* = 0.4; independent *t*-test) and BCVA (0.03 ± 0.07 LogMAR for the customized group vs 0.05 ± 0.07 LogMAR for the control group; *P* = 0.19; independent *t*-test) between the two groups.

### Wavefront aberrations

There was no significant difference in corneal SA before and after surgery at both 4 mm PD (pre, 0.06 ± 0.02 μm; post, 0.05 ± 0.05 μm; *P* = 0.28; paired *t*-test) and 6 mm PD (pre, 0.28 ± 0.09 μm; post, 0.28 ± 0.07 μm; *P* = 0.80; paired *t*-test) for the entire study population. Table 2 shows the postoperative ocular aberration comparisons between the two groups. As expected, all measured HOAs increased when pupil increased from 4 mm to 6 mm. At 4 mm PD, postoperative ocular SA was significantly lower in the customized group than in the control group (0.00 ± 0.03 μm vs 0.02 ± 0.05 μm, respectively; *P* = 0.003), while no significant difference was shown in t-HOA, coma and trefoil (*P* > 0.05); at 6 mm PD, significantly lower t-HOA (0.66 ± 0.17 μm vs 0.74 ± 0.16 μm; *P* < 0.001), SA (0.00 ± 0.12 μm vs 0.12 ± 0.18 μm; *P* < 0.001), coma (0.25 ± 0.13 μm vs 0.33 ± 0.16 μm; *P* = 0.002) and trefoil (0.56 ± 0.16 μm vs 0.62 ± 0.16 μm; *P* = 0.019) were shown in the customized group than in the control group. Moreover, in the customized group, 81% (68/84) of the eyes achieved an ocular SA less than or equal to 0.15 μm postoperatively.

**Table 2:** Postoperative wavefront aberrations (Mean ± SD, μm)

Ocular aberrations		Customized group	Control group	*P* value
4 mm	t-HOA	0.20 ± 0.06	0.21 ± 0.06	0.050
	SA	0.00 ± 0.03	0.02 ± 0.05	0.003^a^
	Coma	0.09 ± 0.05	0.10 ± 0.04	0.066
	Trefoil	0.18 ± 0.06	0.20 ± 0.06	0.088
6 mm	t-HOA	0.66 ± 0.17	0.74 ± 0.16	< 0.001^a^
	SA	0.00 ± 0.12	0.12 ± 0.18	< 0.001^a^
	Coma	0.25 ± 0.13	0.33 ± 0.16	0.002^a^
	Trefoil	0.56 ± 0.16	0.62 ± 0.16	0.019^a^

^a^: indicates statistical significance

### OQAS parameters

Table 3 shows the OQAS parameter comparisons between the two groups. Customized group demonstrated slightly higher MTF cut-off, OV100%, OV20%, OV9%, and lower OSI than the control group with a 4-mm aperture, however, the differences between the two groups were not statistically significant (*P* = 0.54, *P* = 0.65, *P* = 0.73, *P* = 0.64, and *P* = 0.84, respectively). The two groups demonstrated the same in SR (0.15 ± 0.04 in both groups; *P* = 1.00).

**Table 3:** Postoperative OQAS parameters (Mean ± SD)

OQAS parameters	Customized group	Control group	*P* value
OSI	1.47 ± 0.46	1.50 ± 0.71	0.84
MTF cut-off (c/deg)	28.41 ± 8.70	27.50 ± 9.17	0.54
SR	0.15 ± 0.04	0.15 ± 0.04	1.00
OV100%	0.95 ± 0.29	0.93 ± 0.31	0.65
OV20%	0.64 ± 0.21	0.62 ± 0.24	0.73
OV9%	0.36 ± 0.13	0.35 ± 0.13	0.64

*OSI* objective scatter index, *MTF* modulation transfer function, *SR* Strehl ratio, *OV* OQAS value, *c/deg*. cycle per degree

## Discussion

With the advance in wavefront technology, customized selection of aspheric IOLs to compensate individual corneal aberrations has been a trend in the current era of refractive cataract surgery. The aspheric IOLs are designed to compensate the positive corneal SA and thus reduce the ocular SA. The availability of various aspheric IOL designs has enabled the customized selection to precisely achieve a postoperative target ocular SA of zero, which has been considered to be the optimal SA value for producing the best optical quality and visual performance by some researchers [20, 21]. However, there are few controlled studies on optical quality of customized selection with a target SA of zero and the conclusions are inconsistent [13, 14]. The present study aimed to comprehensively compare the postoperative optical quality using a combination of Hartmann-shack wavefront aberrometer and double-pass instrument between customized and random selections of aspheric IOLs, which has been rarely applied in previous studies regarding the customized selection with a target ocular SA of zero.

In this study, no significant differences in postoperative UCVA and BCVA were observed between the two groups, which were consistent with the findings of previous controlled studies related to customized selection [13, 14, 22, 23]. The finding of the mean corneal SA at 6 mm PD was comparable to that in the study by Beiko et al. [2] (0.28 μm vs 0.27 μm), and the unchanged corneal SA after surgery indicated that a bias resulting from the surgical impacts on corneal SA could be avoided. With a 6 mm PD, t-HOA, SA, coma and trefoil were all significantly lower in the customized group, indicating that customized selection reduced the ocular aberrations more effectively than random selection. Moreover, in the customized group, the mean postoperative ocular SA was 0.00 μm, which indeed met the goal of this study, that was, rendering the postoperative ocular SA to be closest to zero. This result was also consistent with the previous studies [11, 12]. In the control group, the postoperative ocular SA was 0.12 ± 0.18 μm, indicating that almost half of the corneal SA remained by using a random selection, which was similar to the result in the study using the same selection strategy by Jia et al. [22] (a residual SA of 0.152 ± 0.151 μm). As is well-known, the coma and trefoil aberrations are mainly related to IOL tilt, decentration and surgical incisions, and the IOL tilt and decentration differ among different types of IOLs [24]. Although the same incisional procedure was used and comparable tilt and decentration were presented in both groups, results demonstrated that coma and trefoil showed significant difference between the two groups when the pupil increased. The only controlled study on customized selection that reported coma and trefoil aberrations was by Nochez et al. [23], which compared a customized group targeting SA to + 0.1 μm with a control group receiving zero SA IOLs only. Results showed similar coma and trefoil in both groups. However, as mentioned, the study design and the IOLs used by Nochez et al. were quite different from the present study, which might lead to the inconsistent results. The unexpected results in this study might be due to the difference of IOL selection strategies in the two groups, which led to imbalance in IOL allocation and thus SA distribution of IOLs between groups. IOLs with various SA values could induce various degree of coma, trefoil and thus t-HOA, even with less noticeable tilt or decentration [16, 25]. However, we had a good reason to apply a random selection strategy as a control, which was the strategy we have been using in actual clinical practice in the past. Overall, the results regarding wavefront aberrations in this study suggested that customized selection of aspheric IOLs based on corneal SA is feasible to achieve a target ocular SA of zero, and a random selection lead to a positive residual SA of 0.12 μm. Customized selection demonstrated a better optical quality in terms of wavefront aberrations at 6 mm PD.

Although lower aberrations demonstrated in the customized group, similar values for all OQAS parameters were shown in both groups in the present study. The results for OVs that represent contrast visual acuity were consistent with Al-Sayyari et al. [13] who found no significant difference in contrast sensitivity between the customized group and the control group. The study by Nochez et al. [23] was the only study applying OQAS system to assess optical quality in customized selection, in which they found significantly better MTF cut-off, OV 100%, OV 20% and OV 9% in the customized group than in the control group. However, their study design was quite different from the present study. They set up the target SA to + 0.1 μm in the customized group and only two SA values (0 μm and − 0.18 μm) of aspheric IOLs were available for selection. The control group received zero SA IOLs only. Eventually, they achieved a mean postoperative ocular SA of 0.085 μm in the customized group and 0.261 μm in the control group, the difference in the mean postoperative ocular SA between groups was 0.176 μm in their study, which was much higher than that in the present study (0.12 μm). This might be the main reason for the inconsistent results between the two studies. As was demonstrated in one of our previous studies, OQAS system could reveal inconsistent outcomes with aberrometers. In that study, we concluded that the optical quality might be overestimated when only aberrations were considered, thus combining the effect of ocular scattering with aberrations by an OQAS system would result in a more accurate assessment of optical quality [16]. Both aberrations and intraocular scatter are known to decrease the quality of the retinal image and therefore the visual performance. Previous evidence suggested that OSI had no significant correlation with SA in pseudophakic eyes (*r* = 0.133, *P* = 0.525), whereas a significantly positive correlation with the total ocular aberrations (*r* = 0.451, *P* = 0.024) [26]. Although the *P* value showed a statistically significance, the correlation coefficient was not large enough to suggest a clinical significance. This may explain why lower aberrations achieved by the customized selection did not lead to an OSI reduction compared to the random selection in the present study. Another previous study investigated the impacts of SA and intraocular scatter on contrast sensitivity, the results demonstrated that contrast sensitivity was reduced less by scatter when SA was present as compared with the cases without SA. They concluded that combined presence of positive SA and scatter could be a mild protective compensatory mechanism reducing the impact that the two factors may have on contrast sensitivity separately [27]. This viewpoint may explain why the overall optical quality interpreted by MTF cut-off, SR and OVs in the control group, though had a larger positive SA, were not worse than the customized group in this study.

In addition, there is currently controversy over the optimal target ocular SA values for an aspheric IOL: 0 μm or + 0.10 μm. Some researchers argued that IOL implantation aiming to reach a target ocular SA to zero could lead to the best optical quality and visual performance [12, 20, 21, 28, 29]. Nevertheless, some researchers argued the same for creating a slightly positive residual ocular SA [22, 23, 30, 31]. Several controlled studies confirmed that both currently argued modes of target ocular SA provided desirable optical quality and visual performance. Specifically, zero SA was more beneficial in mesopic condition while slightly positive SA was more beneficial for near vision [14, 29]. One of the reasons why the overall optical quality of the customized selection did not differ from the random selection might also be that the mean residual ocular SA by a random selection in the present study (+ 0.12 μm) was fairly comparable to + 0.1 μm, which was argued by previous studies to provide a comparable optical quality to that of a zero target ocular SA.

Moreover, as suggested by several previous studies: 1) although SA can be strongly reduced by aspheric IOLs, t-HOA was only slightly reduced. This may be a reason for the unclear results in studies assessing the potential benefit to visual performance of aspheric IOLs [32]; 2) the optimal ocular SA producing the best image quality varied widely between eyes, which was highly associated with the amount of defocus. The amount of optimal ocular SA could be predicted based on other HOAs of the cornea as well. Selection of an aspherical IOL should be customized based on the full spectrum of corneal HOAs, not on SA alone [21]; 3) a study using adaptive optics vision simulator demonstrated that pseudophakic eyes experienced peak contrast sensitivity performance with varying levels of SA when their natural aberrations were present. However, average contrast performance peaked at SA of zero when all HOAs were corrected [20]. These studies indicated that interactions between SA with other HOAs and lower-order aberrations are complicated and not well understood. Customized selection of aspheric IOL implantation based on corneal SA alone may not be an appropriate approach to achieve optimal visual outcomes. Conclusions of these studies may also explain why there was no significant difference in the overall optical quality between customized and random selections of aspheric IOLs in the present study.

Compared with the previous controlled studies, the strengths of the present study were a larger sample size, a more specific customized selection with narrower SA ranges, as well as a more comprehensive and objective assessment for optical quality. There are also some limitations: 1) the focus of the customized design was only on the optimization of postoperative SA; 2) only one target ocular SA value (0 μm) was set up for the customized selection; 3) subjective contrast sensitivity and visual satisfaction were not assessed. Further studies related to customized selection of aspheric IOLs should pay more attention to the full spectrum of preexisting corneal HOAs but not only SA [21]. Further attentions should also be paid to investigate how tilt, decentration and depth of focus impact on the optimization of optical quality in customized selection. It is also necessary to explore the differences in postoperative optical quality by OQAS among customized selection with different target ocular SA values. In addition, visual satisfaction questionnaire should be applied to complement with the objective optical quality assessments.

## Conclusions

In summary, though customized selection of aspheric IOL implantation shows less postoperative ocular aberrations than random selection, they demonstrate similar visual acuity, simulated contrast visual acuity, intraocular scatter and optical quality by using an OQAS system. These results suggest that a customized selection of aspheric IOLs based on corneal SA with a target postoperative ocular SA of zero may not result in an overall optical quality improvement compared to the random selection. In other words, comparably desirable optical quality could be achieved by both customized selection and random selection of aspheric IOL implantation.

## Data Availability

Available upon request from the corresponding author.

## Abbreviations

BCVA: Best corrected visual acuity
D: Diopters
HOAs: Higher-order aberrations
IOL: Intraocular lens
MTF: Modulation transfer function
OQAS: Optical quality analysis system
OSI: Objective scatter index
OV: Optical quality analysis system value
PD: Pupil diameter
PSF: Point spread function
SA: Spherical aberration
SD: Standard deviation
SR: Strehl ratio
t-HOA: Total higher-order aberration
UCVA: Uncorrected visual acuity
